# Physicochemical, Structural, and Digestive Properties of Banana Starch Modified by Ultrasound and Resveratrol Treatments

**DOI:** 10.3390/foods11223741

**Published:** 2022-11-21

**Authors:** Ying Sun, Yang Yang, Lili Zheng, Xiaoyan Zheng, Dao Xiao, Shenwan Wang, Zhengke Zhang, Binling Ai, Zhanwu Sheng

**Affiliations:** 1 Haikou Experimental Station, Chinese Academy of Tropical Agricultural Sciences, Haikou 571101, China; 2 College of Food Science and Engineering, Hainan University, Haikou 570228, China; 3 Haikou Key Laboratory of Banana Biology, Haikou 571101, China

**Keywords:** starch modification, thermal stability, rheological properties, crystallinity, morphology

## Abstract

Ultrasonic treatment combined with resveratrol modification was used to improve banana starch’s solubility, thermal stability, and digestion resistance. The solubility and freeze-thaw stability of the modified starch complex significantly increased. The oil-absorption capacity increased by 20.52%, and the gelatinization temperatures increased from 64.10–73.92 °C to 70.77–75.83 °C. The storage modulus (G′) and loss modulus (G″) increased after ultrasound and resveratrol treatment, and the proportion of viscosity was increased after composition with resveratrol. Additionally, the in vitro digestibility decreased from 44.12% to 40.25%. The modified complexes had release-control ability for resveratrol. X-ray diffraction (XRD) and Fourier transform infrared (FT-IR) spectroscopy demonstrated that complex structures became more compact and organized, whereas crystalline patterns were unchanged. Scanning electron microscopy (SEM) showed that the resveratrol modification caused physical change on the granular surface by creating pores and fissures. The findings can help develop antioxidant functional foods using banana starch.

## 1. Introduction

Bananas are an essential tropical crop and are listed as the fourth largest fruit crop by the Food and Agriculture Organization of the United Nations. Banana starch is the main component of green bananas. It has up to 60% resistant starch (RS), including starch and starchy products that cannot be digested in the human body’s small intestine. Instead, it can be fermented by gut microbiota in the colon [1]. The physiological effects of RS are similar to those of prebiotics and dietary fiber. When RS reaches the colon, it is fermented by the gut microbiota, producing short-chain fatty acids (SCFAs) such as butyric acid, acetic acid, and propionic acid. These SCFAs produced by fermentation are beneficial for the growth of gut bacteria [2]. As banana starch occupies 10–15% of green-banana fruit on banana trees, the byproduct could be a natural resistant starch source. Furthermore, it can be used to develop new healthy foods and improve the banana industry’s economic benefits.

However, native starch has low solubility in cold water, low thermal stability, and low resistance to regeneration. These properties limit the application of starch [3]. Therefore, different technical means are required to modify the starch to improve these properties for better application. Ultrasonication technology can transmit the energy to starch granules via cavitation. Cavitation includes the formation, growth, and rapid collapse of microbubbles, forming micro-jets, shearing force, local heating, and breaking of the C-C bond of macromolecules. Collectively, these effects can change the structural property of starch [4]. For example, ultrasonic treatment was reported to significantly increase the water solubility of quinoa [5]. Nie et al. [6] found that ultrasonication reduced starch retrogradation and improved the intrinsic viscosity of potato-starch paste. Additionally, it was found that ultrasonication improved the gelatinization temperatures of corn starch [7]. Han et al. [8] revealed that ultrasound treatment elevated the RS content of pea starch. Although these modifications were observed with other starch products, the change in structure and physicochemical properties of banana starch after ultrasound treatment has not been reported.

Resveratrol (trans-3,5,4′-trihydroxystilbene, Res), a natural polyphenol, is abundant in red wine, peanuts, grape, and tomato peel. The benefits of resveratrol are its neuroprotective, anticancer, antioxidant, and anti-inflammatory properties [9]. However, Res’ water solubility, absorption, and bioavailability are very low, limiting its application in the food and medicine fields. Some polysaccharides, like chitosan [10], pectin [11], and cellulose [12], have been used for encapsulating it to form biopolymer particles to protect and deliver polyphenols. Due to its non-toxicity, harmlessness, and biocompatibility, starch is also a material for encapsulation that can form and transport a matrix of polyphenols to maintain polyphenol stability during food manufacture and digestion [13]. Polyphenols can complex with starch molecules via non-covalent interactions (such as hydrogen bond, hydrophobic interaction, or van der Waals force) and compete with starch molecules for the active sites of amylases. In doing so, this increases the anti-digestion ability and thermal stability of the starch [14]. Additionally, persimmon tannin improved the gelatinization temperature and retarded the retrogradation of maize starch [15]. Barros et al. [16] found that the RS content was increased in corn starch after adding sorghum phenolics. Furthermore, Shen et al. [17] revealed that resveratrol was a competitive inhibitor for α-amylase.

Nevertheless, single polyphenol or ultrasonic treatment could affect the properties of starch. The combined effect of ultrasonic treatment with polyphenols on the properties of banana starch has not been reported. In this study, ultrasonic treatment combined with resveratrol modification was used to improve banana starch’s solubility, thermal stability, and digestion resistance. In addition, the physicochemical, in vitro digestibility, and release properties of resveratrol from the complexes were characterized. Furthermore, the mechanism of the effects of ultrasound treatment and resveratrol complexation on the properties of banana starch was investigated. Collectively, the results of this study could provide a new approach to developing functional foods using green-banana byproducts.

## 2. Materials and Methods

### 2.1. Materials and Chemicals

Green banana (Musa AAA Cavendish subgroup cv. Nan tian huang), with a pericarp color index of 1 (according to the colorimetric card provided by SH Pratt (Luton, UK)), was provided by the Experimental Base of the Chinese Academy of Tropical Agricultural Sciences. Resveratrol was purchased from Shanghai Aladdin Bio-technology Co., Ltd. (Shanghai, China). Saliva α-amylase, pepsin, trypsin, amyloglucosidase, and porcine bile salt were purchased from Sigma-Aldrich Trading Co., Ltd. (Shanghai, China). The D-glucose assay kit (GOPOD Format K-GLUK) was purchased from Shanghai Megazyme Bio-technology Co., Ltd. (Shanghai, China). Other chemicals used in this experiment were of analytical grade.

### 2.2. Extraction of Banana Starch

Banana starch was extracted according to the previous procedure [18]. After green bananas were washed, their peels were removed. Then, the banana flesh was cut into 2 mm slices and immediately placed in the color-protection solution (20 g/L citric acid and 2 g/L ascorbic acid) for 15 min, followed by homogenization in a blender (Zhejiang Supor Co., Ltd., Zhejiang, China) with ultrapure water (1:2, *w*/*v*) for 2 min. The homogenized mixture was centrifuged (Sorvall LYNX, Thermo Fisher Technology Co., Ltd., Shanghai, China) at 5000 rpm for 10 min. The sediments were isolated and dried in an oven (CS101-ABN, Yongsheng Test Equipment Co., Ltd., Chongqing, China) at 45 °C for 6 h. The dried banana starch was ground into powder by a mortar (Beijing Zhongke Aobo Technology Co., Ltd., Beijing, China) and screened through a 100 mesh sieve (Shanghai Merxi Scientific Instrument Co., Ltd., Shanghai, China). The starch was stored in a brown reagent bottle and put in the freezer (Haier Co., Ltd., Qingdao, China) at 4 °C for further use. The prepared banana starch was native starch and named NS.

### 2.3. Ultrasonic Treatment

The starch was dispersed in ultrapure water (1.0%, *w*/*v*) and treated using an ultrasonic processor (20 kHz, JY 92-IIN Ningbo Scientz Biotechnology Co., Ltd., Ningbo, China) for 10 min in a pulsed mode. The ultrasound amplitudes were selected as 40%, 60%, 80%, and 100% and marked as U_40_S, U_60_S, U_80_S, and U_100_S, respectively. The sample was placed in an ice bath when operating to prevent the slurries’ temperature from rising. Then, the slurries were centrifuged (Sorvall LYNX, Thermo Fisher Technology Co., Ltd., Shanghai, China) at 5000 rpm for 10 min, and the sediments were dried (CS101-ABN, Yongsheng Test Equipment Co., Ltd., Chongqing, China) at 45 °C for 6 h and ground by a mortar (Beijing Zhongke Aobo Technology Co., Ltd., Beijing, China).

### 2.4. Preparation of Green-Banana-Starch–Resveratrol Complexes

Banana-starch–resveratrol complexes were prepared referring to Gao et al. [14] with some differences. Briefly, banana starch treated by ultrasound or untreated (10 g) and resveratrol (1.0 g, 10% based on starch weight) were suspended in 200 Ml of 30% ethanol solution with a stirring machine (HJ-6A, Jiangsu Jinyi Instrument Technology Co., Ltd., Jiangsu, China) at 150 rpm and 70 °C for 1 h. The suspensions were subsequently cooled and centrifuged (Sorvall LYNX, Thermo Fisher Technology Co., Ltd., Shanghai, China) at 5000 rpm for 10 min. The sediments were washed with a 50% ethanol solution to remove uncombined resveratrol, then centrifuged at 3000 rpm for 15 min. The resin step was repeated 3 times. Finally, the sediments were freeze dried (SCIENTZ-12N/A, Ningbo Xinzhi Biotechnology Co., Ltd., Ningbo, China) for 12 h and ground. The final samples were named Res-NS, Res-U_40_S, Res-U_60_S, Res-U_80_S, and Res-U_100_S.

### 2.5. Complex Index

The complex index (CI) was used to determine the degree of starch–resveratrol complex formation [19]. The starch–resveratrol complex (1 g) was blended with 25 mL of ultrapure water in a centrifuge tube. Then, the suspensions were heated by an induction cooker (C22-RT22E01, Midea Group Co. Ltd., Guangzhou, China) in a boiling-water bath for 2 min until the starch was gelatinized entirely. The dispersion was centrifuged (Sorvall LYNX, Thermo Fisher Technology Co., Ltd., Shanghai, China) at 10,000 rpm for 15 min after natural cooling to 25 °C. Then, 300 μL of supernatant were thoroughly mixed with 5 mL of ultrapure water (Milli-Q Reference, Merck Chemical Technology Co., Ltd., Shanghai, China) and 1 mL of iodine solution (1.3% (*w*/*w*) I_2_ and 2.0% (*w*/*w*) KI in ultrapure water) in a tube. Iodine-binding-capacity (IBC) values of the sample and control (without resveratrol) were detected at 690 nm. The CI was calculated using Equation (1):

(1) $$\mathrm{CI}\left(\%\right)=\left({\mathrm{IBC}}_{\mathrm{reference}}{\;-\;\mathrm{IBC}}_{\mathrm{sample}}\right)/{\mathrm{IBC}}_{\mathrm{reference}}\times 100$$

where CI (%) is the complex index, IBC_reference_ is the absorbance value of the reference, and IBC_sample_ is the absorbance value of the sample.

### 2.6. Physicochemical Properties

#### 2.6.1. Solubility and Swelling Power

Guo et al. [20] was referenced for the solubility (S) and swelling power (SP) of starch. In a centrifuge tube, the sample was mixed with ultrapure water to prepare the suspensions (2.0%, *w*/*v*). The suspensions were heated at 50, 60, 70, 80, and 90 °C in a water-bath shaker (SWB-2000, Tianjin Aoxenes Instrument Co., Ltd., Tianjin, China) for 30 min. The mixture was centrifuged (Sorvall LYNX, Thermo Fisher Technology Co., Ltd., Shanghai, China) at 4000 rpm for 10 min after natural cooling. The sediments were dried (CS101-ABN, Yongsheng Test Equipment Co., Ltd., Chongqing, China) at 105 °C to a constant weight. The S and SP were calculated as Equations (2) and (3), respectively:

(2) $$S\;\left(\%\right){=W}_{1}\;/\;W_{0}\times \;100$$

(3) $$\mathrm{SP}\;\left(g\;/\;g\right){=W}_{2}\;/\;W_{0}$$

where W_1_ is the constant weight after drying, W_0_ is the initial weight of the starch sample, and W_2_ is the weight of the swollen starch.

#### 2.6.2. Oil-Absorption Capacity

The oil-absorption capacity (OAC) of the starch–resveratrol complexes was measured based on Wang et al. [21]. Starch (1.0 g) and oil (20 mL) were blended in a weighted centrifuge tube and shaken in a water-bath shaker (SWB-2000, Tianjin Aoxenes Instrument Co., Ltd., Tianjin, China) for 30 min at 25 °C. After that, the suspension was centrifuged (Sorvall LYNX, Thermo Fisher Technology Co., Ltd., Shanghai, China) at 5000 rpm for 15 min. The supernatant was removed completely. Following this step, the centrifuge tube and the precipitate were weighed. The OAC values of the samples were obtained with Equation (4):

(4) $$\mathrm{OAC}\;\left(g\;/\;g\right)=\left(m_{2}\;-{\;m}_{1}\right)\;/\;m_{1}$$

where m_1_ (g) and m_2_ (g) are the weight of original starch and the precipitate, respectively.

#### 2.6.3. Freeze-Thaw Stability

The freeze-thaw stability of the samples was analyzed based on the report of Zheng et al. [22]. Briefly, starch (1.0 g) and ultrapure water (16 mL) were blended in a weighted centrifuge tube. Then, the suspension was heated (C22-RT22E01, Midea Group Co. Ltd., China) in a boiling-water bath for 30 min. Starch gels were frozen at −20 °C for 24 h in a freezer (Haier Co., Ltd., Qingdao, China). Then, the samples were thawed at room temperature for 6 h and centrifuged (Sorvall LYNX, Thermo Fisher Technology Co., Ltd., Shanghai, China) at 4500 rpm for 15 min. The supernatant was abandoned and the remaining precipitate was weighed. The freeze-thaw cycle was repeated three times. Syneresis reflects the stability of the freeze-thaw (Equation (5)).

(5) Syneresis (%)=(W / M) × 100

where W (g) is the weight of supernatant, and M (g) is the weight of original dry starch.

#### 2.6.4. Analysis of Thermal Properties

Thermal properties of starch samples were determined using differential scanning calorimetry (DSC7000, Hitachi., Tokyo, Japan). Three milligrams of starch sample and ultrapure water (1:3) were mixed in DSC pans. Then, the sealed pans were equilibrated at 25 °C for 24 h. The samples were heated from 20 to 100 °C at 10 °C/min during DSC testing, and an empty pan served as the control. The onset temperature (T_o_), peak temperature (T_p_), end-set temperature (T_c_), and enthalpy change (ΔH) of each sample were determined.

### 2.7. Rheological Properties

Samples were accurately weighed to 1.0 g and ultrapure water was added to prepare 5% suspension, then gelatinized for 30 min in a boiling-water bath (C22-RT22E01, Midea Group Co. Ltd., Guangzhou, China) and naturally cooled to 25 °C. A total of 1 mL of gelatinized starch paste was absorbed, and steady-state shear and dynamic oscillation (frequency sweep) analyses of the sample were carried out by a rotary rheometer (DHR-1, Waters Co., Ltd., Milford, MA, USA).

#### 2.7.1. Steady Shear Analysis

The starch paste was placed on the rheometer plate using a parallel plate with a diameter of 40 mm and a gap of 1 mm. The sample’s shear stress (Pa) was measured with a shear rate of 0.1–200 s^−1^ at 25 °C. The data were fitted into Equation (6).

(6) $$\delta =K\;\cdot {\;\gamma }^{n}$$

where δ is shear stress (Pa), K is the consistency coefficient (Pa·s^n^), and n is the flow-behavior index.

#### 2.7.2. Dynamic Oscillatory Analysis

After the operation in Section 2.7.1, the gel was equilibrated for 120 s at 25 °C. Then, it was swept at a frequency of 0.1–20 Hz with a sweep stress of 2% at 25 °C. The storage modulus (G′), loss modulus (G″) and damping factor (tan δ = G″/ G′) were recorded.

### 2.8. Determination of In Vitro Digestibility

In vitro digestibility of the starch samples was based on Ye et al. [23]. Mouth, gastric, and small-intestine phases comprise a three-step simulated gastrointestinal-tract (GIT) system. The simulated saliva fluid (SSF), simulated gastric fluid (SGF), and simulated intestinal fluid (SIF) were similar to the method of Ye et al. [23].

For the mouth phase, 50 mg of the starch samples were added to 10 mL of SSF containing α-amylase (75 U/mL, 37 °C). Then, the suspension was adjusted to pH 6.8 and shaken in a water bath (37 °C, 100 rpm) for 2 min.

For the gastric phase, the SGF (37 °C, 20 mL) including pepsin (2000 U/mL) was mixed with the mouth-phase sample and adjusted to pH 3.0. Then, the suspension was shaken at 37 °C for 2 h at 100 rpm.

For the small-intestine phase, the suspension (20 mL) obtained from the gastric phase was adjusted to pH 7.0. After that, 10 mL of SIF, 5.0 mL of a pancreatin (800 U/mL) and amyloglucosidase (15 U/mL) solution consisting of SIF, and 3.5 mL of bile extract (53.57 mg/mL) were mixed with the sample. Then, 0.15 mL NaOH (0.1 M) were adjusted to pH 7.0 and mixed with 1.35 mL of water to make up 20 mL. The suspension was shaken under the same conditions for an additional 150 min.

The sample (0.5 mL) was taken after 2 min reaction time in the mouth phase (S2), after 30 min and 120 min reaction times in the gastric phase (G30 and G120), and after 10, 20, 60, 90, 120, and 150 min reaction times in the small-intestine phase (I10-I150) to measure the hydrolysis degree of the starch at each time. The content of glucose was determined via a D-glucose assay kit. Starch hydrolysis (%) was calculated by Equation (7):

(7) $$\mathrm{Starch}\;\mathrm{hydrolysis}\;\left(\%\right)=0{.9\;\times G}_{h}\;/\;S_{i}\times \;100$$

where S_i_ is the original amount of starch, and G_h_ is the amount of glucose produced. A constant of 0.9 was used to convert the molar mass from glucose to anyhydroglucose.

Equation (8) was used to describe the kinetics of starch hydrolysis [24]:

(8) $$C_{t}{=C}_{\infty }\left({1\;-\;e}^{-\mathrm{kt}}\right)$$

where C_t_ (%) is the percentage of digestion at time t (min), C_∞_ (%) is the final percentage of digestion, k (min^−1^) is the starch-digestion-degree coefficient, and t is the reaction time. The logarithm-of-slope (LOS) analysis was determined through Equation (9) [25]:

(9) $$\ln\;\left({\mathrm{dC}}_{t}\;/\;\mathrm{dt}\right)=-\mathrm{kt}+\ln\;\left(C_{\infty }k\right)$$

The contents of rapidly digestible starch (RDS), slowly digestible starch (SDS), and RS in the samples were determined with Equations (10)–(12) [26]:

(10) RDS (%)=(G20 − F) × 0.9 / T× 100

(11) SDS (%)=(G120 − G20) × 0.9 / T× 100

(12) RS (%)=1 − [RDS (%)+SDS (%)]

where F is the total amount of free glucose at the start of the intestinal digestion phase, and T is the original total amount of starch.

### 2.9. Structural Characterization

#### 2.9.1. Fourier Transform Infrared (FT-IR) Spectroscopy

The FT-IR spectra of the starch samples were obtained through an IR PRE-STIGE-2 (Shimadzu, Kyoto, Japan) in wavenumbers of 400–4000 cm^−1^. The samples were blended with KBr powder and pressed into round tablets. They were then measured by FT-IR spectroscopy.

#### 2.9.2. X-ray Diffraction (XRD) and Relative Crystallinity (RC)

An X-ray diffractometer (D8 ADVANCE, Malvern Instruments, Malvern, UK) under 40 kV and 40 mA with Cu Kα radiation was used to analyze the samples’ crystalline structures. Data were obtained between 4 and 40° (2θ) at a rate of 4°/min. The RC values of the samples were determined with Equation (13):

(13) $$\mathrm{RC}\;\left(\%\right){=I}_{C}\;/\;\left(I_{C}{+I}_{A}\right)\times \;100$$

where I_C_ and I_A_ refer to the cumulative diffraction intensity of the crystallized region and the amorphous region, respectively.

#### 2.9.3. Morphological Analysis

Morphology of the samples was detected by a scanning electron microscope (JSM-6360LV, 139; JEOL, Tokyo, Japan). The samples were coated with gold and measured at an accelerating voltage of 20 kV.

### 2.10. In Vitro Resveratrol Release from Starch–Resveratrol Complex

The release rate of resveratrol from Res-NS and Res-U_60_S during digestive-tract digestion was determined by a dialysis method based on Mohammadian et al. [27] and Chi et al. [28] with some adjustments. The starch–resveratrol complexes were placed in a dialysis bag and mixed with 5 mL digestive juice. Then, the dialysis bag was put in a beaker with 200 mL of the release medium. The release medium consisted of 60 mL ethanol and 140 mL enzyme-free digestive juice. They were shaken at 37 °C and 100 rpm for 150 min. At each sampling time, 1 mL of the release medium was absorbed and 1 mL fresh medium was added to keep the total volume constant. The amount of resveratrol released was detected by a UV-vis spectrophotometer (UV-1800, Shimadzu, Kyoto, Japan) at 306 nm. In order to investigate the in vitro release mechanism of resveratrol from the complexes, the experimental results were fitted using the following kinetic-release models:

(14) $$\mathrm{Zero}-\mathrm{order}\;\mathrm{model}:\;M_{t}\;/\;M_{\infty }{=k}_{0}t$$

(15) $$\mathrm{First}-\mathrm{order}\;\mathrm{model}:\;\ln\;\left({1\;-M}_{t}\;/\;M_{\infty }\right){=-k}_{1\;}t$$

(16) $$\mathrm{Higuchi}\;\mathrm{model}:\;M_{t}\;/\;M_{\infty }{=k}_{H}{\;t}^{1/2}$$

(17) $$\mathrm{Ritger}–\mathrm{Peppas}\;\mathrm{model}:\;M_{t}\;/\;M_{\infty }{=k}_{P}{\;t}^{n}$$

where M_t_/M_∞_ is the cumulative release rate of resveratrol at t time (%); k_0_, k_1_, k_H_, and k_P_ are the release-rate constants; and n is a dimensionless number.

### 2.11. Statistical Analyses

All tests were repeated in triplicate. The experimental data were analyzed by SPSS 23.0 using analysis of variance (ANOVA) and Tukey’s multiple-range test at the 5% significance level.

## 3. Results and Discussion

### 3.1. Effect of Ultrasound Treatment on CI

**Figure 1:**
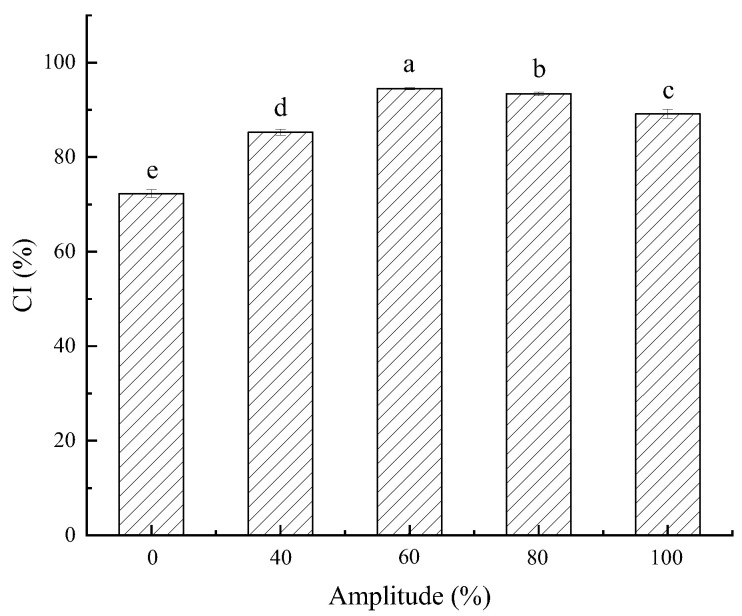
CI (%) after different ultrasonic-amplitude processing. Lowercase letters indicate a statistically significant difference within the group (*p* ≤ 0.05). Error bars represent standard deviation (n = 3).

Changes in the CI values of the starch–resveratrol complexes with different ultrasound-amplitude treatments are presented in Figure 1, and these values reflect the ability of banana starch and resveratrol to form a starch–polyphenol complex. The CI after the ultrasound treatment was larger than that of the untreated one. Hence, a higher CI value suggests more starch–polyphenol binding formed between the banana starch and resveratrol. Therefore, the ultrasound treatment promoted the starch–polyphenol complex formation and suggests that ultrasonication made the starch granules disintegrated, and the molecular scission of the starch chains increased the probability of contact between the resveratrol and starch molecules [29].

Figure 1 suggests that the CI values initially increased and then decreased at a higher ultrasound amplitude (>80%). The results reflect that the ultrasound power at a specific range was favorable to the formation of the complex because ultrasound irradiation could promote the big starch granules to fracture into small pieces in the starch slurry. The CI values gradually reduced as the ultrasound amplitude continuously increased. A high ultrasonic power could make the tiny starch fragments reunite as new granules or get stuck on the surface of the banana-starch granules. It may have reduced the contact between the resveratrol and starch [4]. Considering that the higher CI had more nutrition, we chose the highest CI, so the ultrasound treatment at 60% amplitude was the best condition. The following results and discussion take into consideration only this treatment.

### 3.2. Characterization of Physicochemical Properties

#### 3.2.1. S and SP

**Figure 2:**
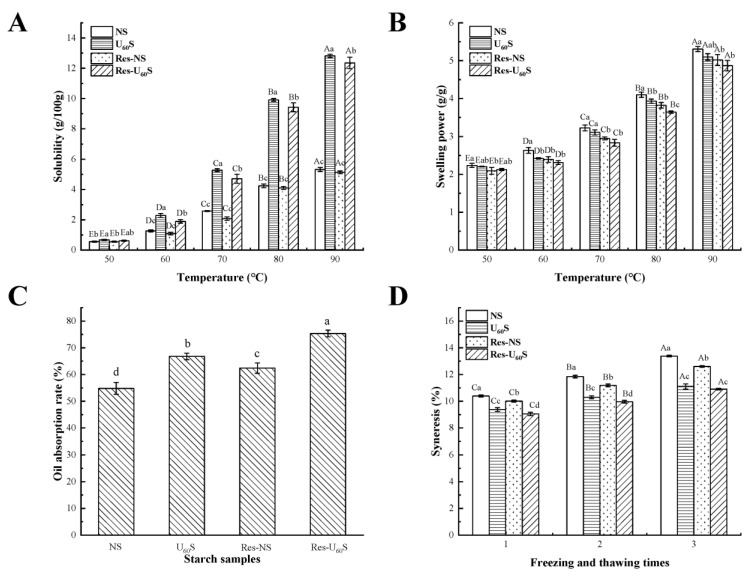
Solubility and swelling power of banana starch affected by ultrasonication and resveratrol (**A**,**B**). (**C**) Oil-absorption capacity of different starch samples. (**D**) The changes in syneresis of different banana starches. Lowercase letters indicate a statistically significant difference within the group (*p* ≤ 0.05), and uppercase letters indicate a statistically significant difference between groups (*p* ≤ 0.05). Error bars represent standard deviation (n = 3).

The S and SP of the starch reflect the capacity of dissolution and water absorption, respectively. The S and SP of all samples in the range of 50–90 °C are exhibited in Figure 2A,B. Here, all samples’ S and SP gradually increased from 50 to 90 °C. Higher temperatures may increase the migration and leaching of amylose from the surface of crystals. Therefore, the S was increased [3]. Compared to NS, the S of U_60_S significantly increased whereas its SP gradually decreased. This could have been caused partly by the structural change and the facial cavities after ultrasonic treatment, thereby providing channels, contributing to water penetration, and diffusing into granules. These findings support the result of Hu et al. [30] and Monroy et al. [31]. Ultrasonic treatment could also have resulted in molecular depolymerization and increased the ratio of short chains. Starch could have had a better hydration tendency [32]. The decrease in the SP could indicate an increase in the order or interactions between starch molecules. It decreased the degree of hydration of amorphous regions and inhibited the swelling and leaching of molecules [33]. Whether NS or U_60_S, the starches’ S and SP were reduced after being treated with resveratrol (Res-NS and Res-U_60_S). The decrease in solubility could have been due to the cross-linking promoted by resveratrol in starch molecules that compacted the starch–resveratrol complex’s crystal pattern [14], and the SP was mainly related to amylopectin, which could have formed the crystalline structures of the starch [34]. Furthermore, the arrangement of amylopectin may have caused it to be more compact and uniform. Hence, the SP of the banana starch was reduced.

#### 3.2.2. Oil-Absorption Capacity

The oil-absorption capacity (OAC) of starch could be an index for the process of oily starch-based food products. The OAC values of different starch samples are shown in Figure 2C. After ultrasonic treatment, the starch OAC significantly increased from 54.81% to 66.79%. This increase could be ascribed to the physical change by ultrasound treatment, such as pores, cracks, and depressions on the surface, which facilitated the diffusion of fat [35]. In addition, the loose and hydrophobic structure of amylose may have increased OAC [36], with similar results reported by Wang et al. [21]. Compared with NS, adding resveratrol could have significantly increased the OAC of starch. Likewise, the OAC of Res-U_60_S was higher than that of U_60_S. This effect may have been due to resveratrol acting as a mediator to form a starch–resveratrol–starch network structure by improving the OAC of starch.

#### 3.2.3. Freeze-Thaw Stability

The content of separated water during the freeze-thaw process indicated a trend of starch retrogradation and was evaluated by syneresis [37]. Figure 2D demonstrates the syneresis of samples during the three cycles. These findings indicate that ultrasonication enhanced the freeze-thaw stability of the samples. Additionally, the syneresis of Res-NS and Res-U_60_S were significantly reduced when in a complex with resveratrol, indicating that resveratrol could have enhanced the freeze-thaw stability of starch. With the cycles increasing, either ultrasound or resveratrol treatment could have decreased the syneresis. Okonkwo et al. [36] reported that the breaking of starch chains in the amorphous region caused significant reorganization of chain fragments under ultrasonic treatment. During the freeze-thaw cycle, the –OH groups of resveratrol bind with starch molecules. One resveratrol molecule can simultaneously interact with multiple starch molecules and act as a cross-linking agent. This interaction may have enhanced the amorphous molecular order and pack the semi-crystalline regions more tightly [13]. Hence, less water leached out from complex molecules after the freeze-thaw cycles than NS and U_60_S.

#### 3.2.4. Thermal Characteristics

The thermal characteristics of different starches were determined by DSC. The thermal parameters (T_o_, T_p_, and T_c_) and the enthalpy change (ΔH) are shown in Table 1. Banana starch treated with ultrasonication (U_60_S) had significantly higher onset, peak, and conclusion gelatinization temperatures than those of NS. The enthalpy change (ΔH) of U_60_S was also higher than that of NS. The ratio of amylose and amylopectin affected the gelatinization of the starch. Ultrasonic treatment could have promoted amylose to seep out from the inner structure of the starch and partially gelatinize the surface of the starch under high temperatures. This may have prevented further water penetration and caused the thermal energy to increase [34]. We found that the ΔH of starch complexing with resveratrol (Res-NS and Res-U_60_S) increased compared with the samples without resveratrol. This finding suggests that resveratrol could increase the energy requirement in starch gelatinization by acting as a bridge to increase the recombination of starch molecules and crystallization order. Thus, more energy is required to reduce the dense crystalline structure of starch [38].

### 3.3. Steady Shear Analysis

**Figure 3:**
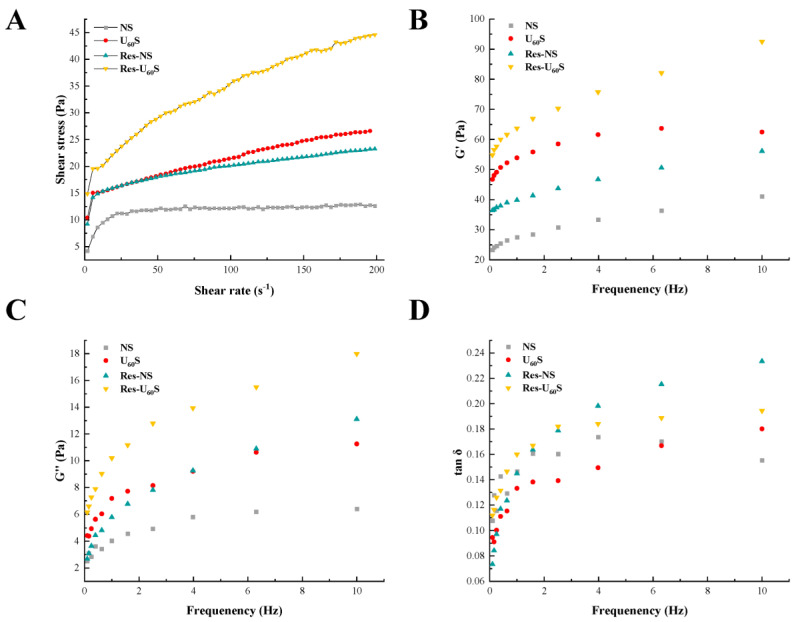
Flow curves (**A**), storage modulus (G′, (**B**)), loss modulus (G″, (**C**)), and tan δ (**D**) of starch samples as a function of oscillation frequency.

The effects of different treatments on banana starch’s steady-state rheological properties are displayed in Figure 3A, and the results were fitted into the power-law equation (Table 2). In the selected range of shear rate, all the starch samples’ shear stress increased with the increase in shear rate, showing different degrees of the convex shear-stress axis. All of the n values showed non-Newtonian pseudo-plastic behavior (n < 1). After ultrasonic treatment, the starch molecular structure was confused and the starch chains were re-associated, causing the intermolecular binding force to increase to form a stable structure [39]. Therefore, the viscosity of molecular flow and shear stress increased. The complexation of resveratrol increased the K and n, which was caused by the dense network structure formed via the interaction between banana starch and resveratrol [14].

### 3.4. Dynamic Oscillatory

The dynamic rheological properties of samples are shown in Figure 3B–D. The G′ and G″ of natural starch (NS) and modified starch increased with the increasing frequency, and G′ was larger than G″. When the frequency was constant, the G′ and G″ of starch treated by ultrasound (U_60_S) were higher than those of NS. In comparison, tan δ (G″/G′) was smaller than that of NS, showing that U_60_S had stronger gel strength than NS. Generally speaking, when the G′ increases, the tan δ decreases, which means polymer cross-linking occurs [40]. Therefore, the enhancement of the network structure of U_60_S may have been due to permanent cross-linking in the sample. Monroy et al. [31] revealed that ultrasound treatment could break macromolecular starch chains and the short polymerization links reassociated by hydrogen bonds, forming a more viscoelastic three-dimensional network. All the samples’ tan δ values were below 1, indicating the formation of a weak gel system that had “solid-like” characteristics [23].

The G′ and G″ of starch paste increased markedly after complexation with resveratrol. The resveratrol acted as a bridge between starch molecules, and it was beneficial for forming a starch or starch–resveratrol–starch network, causing the viscoelasticity of the starch paste to increase. Chi et al. [28] revealed a similar result when gallic acid was compounded with rice starch. However, the G′ and G″ of rice starch decreased when the addition of gallic acid was high. The G′ and G″ of rice starch were also reduced after complexation with tea polyphenols [41]. These differences could be related to the treatment conditions, the species of starch and polyphenols, and the additional amounts of polyphenols. The increased tan δ indicates that the proportion of viscosity and fluidity of the complex increased when modified by resveratrol. The paste was inclined to viscous fluid.

### 3.5. In Vitro Digestibility

**Figure 4:**
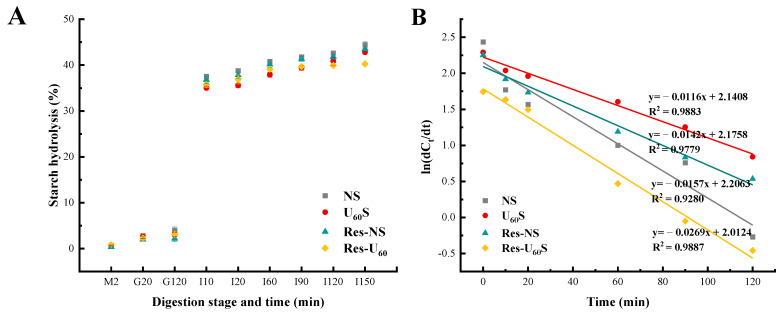
(**A**) Changes in starch hydrolysis (%) of different samples in in vitro starch digestion. Error bars represent standard deviation (n = 3). Key: M = simulated mouth-digestion stage, G = simulated gastric-digestion stage, and I = simulated small-intestinal-digestion stage. The numbers following M, G, and I represent the digestion time in minutes for each stage. (**B**) LOS plots of samples.

The hydrolysis patterns and logarithm of slope plots of banana starches after different treatments are shown in Figure 4. The hydrolysis of samples was determined using the simulated GIT method. The four starch samples had low hydrolysis levels (<10%) in the mouth and gastric phases. However, in the small-intestine phase, the hydrolysis of all the samples increased sharply. The final degree of digestion among the four starch samples followed the order of NS > Res-NS > U_60_S > Res-U_60_S.

The k and C_∞_ of hydrolysis were calculated using a first-order model through the digestion curve (Table 3). The ultrasound and resveratrol treatments significantly decreased C_∞_, although the tendency of k did not entirely agree with C_∞_. The starch digestion at the initial stage depends on the hierarchical structure, whereas C_∞_ is affected by enzyme activity. Therefore, the k and C_∞_ were influenced by the combined effects, i.e., the sample structure and the resveratrol inhibition against amylases [28].

The modified starch can be classified into RDS, SDS, and RS based on different digestion levels. From Table 3, the contents of RDS and RS of U_60_S were apparently (*p* ≤ 0.05) larger than that of NS, whereas SDS content was significantly lower. This agreed with the report of Han et al. [8]. The increase in RDS could be explained by ultrasound treatment, which caused the degradation of the starch chain and formed cracks and holes on the granule surface, increasing the accessibility of enzymes [42]. When the resveratrol was complexed, the starch gels were reassembled, forming higher short-range orders and more compact structures [28]. Moreover, Li et al. [43] reported that the heating–gelatinization–cooling process may increase the content of RS. Hence, the digestibility of banana starch was significantly reduced.

### 3.6. Structural Characterization of Starch–Resveratrol Complexes

#### 3.6.1. Short-Range Ordered Structure

**Figure 5:**
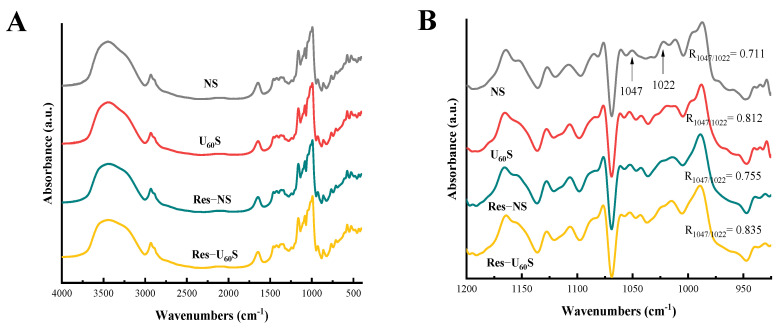
FTIR (**A**) and ATR-FTIR (**B**) spectra of different starch samples.

The ATR-FTIR spectra of the samples are exhibited in Figure 5. All the starch samples had three major peaks at 3000–4000 cm^−1^, 2925 cm^−1^, and 1654 cm^−1^, corresponding to O-H, C-H, and -CH_2_ stretching vibrations, respectively [44]. There was no new peak in the other three samples compared to NS. These findings indicate that no new functional groups or chemical bonds were produced during the ultrasound and resveratrol treatments. Hence, the resveratrol interacted with starch mainly via non-covalent bonds, which is supported by findings of Han et al. [8] and Su et al. [45]. The band ranging from 1200–900 cm^−1^ is the fingerprint-structure region of the starch. The absorption peaks at 1047 and 1022 cm^−1^ are related to the crystalline and amorphous structures of the starch, respectively. The absorbance ratio of 1047/1022 cm^−1^ (R_1047/1022_) reflects the degree of short-range order on the surface of the starch granules [44]. Banana starch modified by ultrasonication (U_60_S) had a higher R_1047/1022_ than that of NS. Compared with NS and U_60_S, R_1047/1022_ of Res-NS and Res-U_60_S increased after adding resveratrol. The increase in R_1047/1022_ indicates that ultrasound and resveratrol treatments could increase the short-range order of samples and can be explained by some of the dispersed starch chains being tightly packed in amorphous regions after ultrasonic treatment. In contrast, resveratrol was bound to the starch chains through non-covalent interactions and acted as a cross-linking agent that enhanced the molecular organization [13,35].

#### 3.6.2. Long-Range Ordered Structure

**Figure 6:**
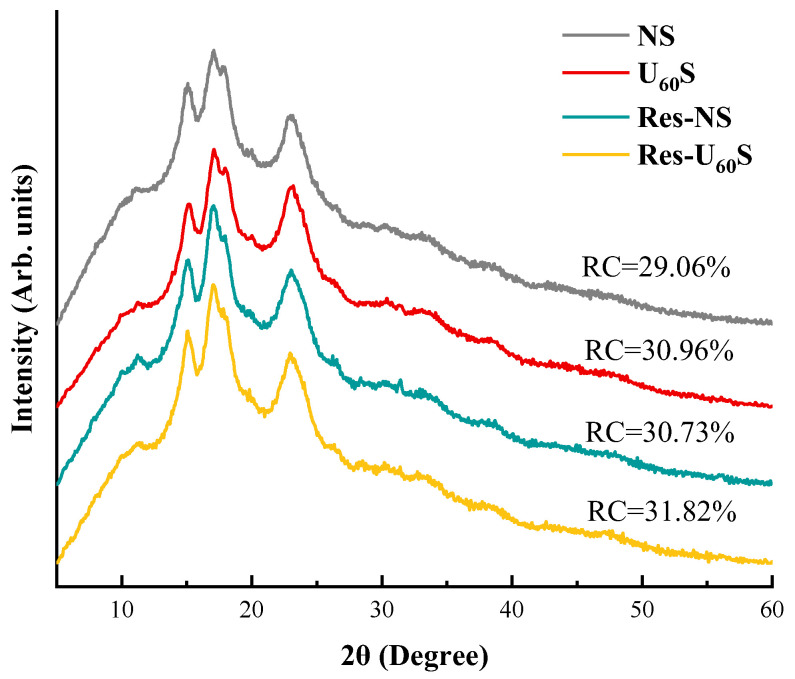
X-ray diffraction pattern and relative crystallinity (RC) values of four samples.

X-ray diffraction patterns were used to characterize the crystal structure of the starch samples. The crystalline features of the different treatments are displayed in Figure 6. All the starches showed a typical C-type crystalline arrangement and diffraction peaks at approximately 15°, 17°, and 23° (2θ). This indicates that the ultrasound and resveratrol treatments did not alter the crystalline type of the samples, which is similar to previous results [14,46]. Additionally, the absence of diffraction peaks at 7°, 13°, and 20° (2θ) illustrates that no V-type inclusion complex was formed between resveratrol and banana starch, so resveratrol did not occupy the cavity of the banana-starch helix. The driving force of the V-type complex formation is the hydrophobic interaction in the starch helical cavity. The lack of a V-type complex could have been due to the large size or the insufficient hydrophobicity of the phenolic [47]. As the molecular size of resveratrol is small, it could have been caused by the lack of hydrophobicity of resveratrol.

The RC of the sample increased from 29.06% to 30.96% after ultrasonic treatment. Compared to crystalline regions of starch, ultrasonic treatment significantly affected the amorphous regions. This agrees with a previous study [48]. The arrangement of linear polymeric chains in the amorphous region was broken by ultrasonic treatment. The packing arrangements of amylose–amylose and amylose–amylopectin could have increased the sample’s crystallinity if the arrangements were tighter [49,50,51]. The RC of starch complexing with resveratrol (Res-NS and Res-U_60_S) increased compared with the samples without resveratrol (NS and U_60_S, respectively). Resveratrol bound to hydrogen groups in the α-D-glucose of amylose or amylopectin chains in starch granules. It then stacked aromatic residues with the amylopyranose ring through 2–3 CH-π bonds and caused the amorphous molecules to become more ordered with an increase in RC [13,52]. This is in accordance with the DSC and FTIR analysis.

#### 3.6.3. Morphological Changes

**Figure 7:**
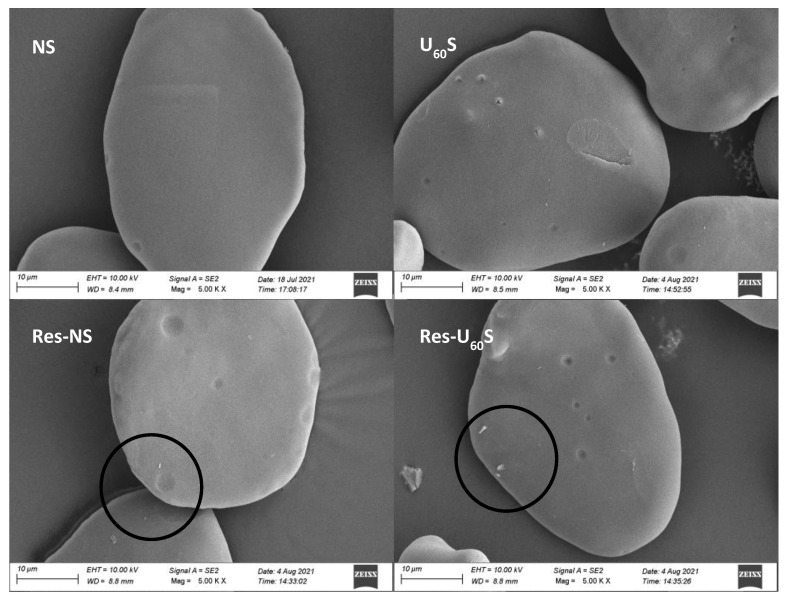
Scanning electron micrographs of samples.

The morphological changes in the modified banana-starch granules by ultrasonication and resveratrol treatment are exhibited in Figure 7. The NS granules showed oval and irregular shapes. The dual modification did not change the outline of the granules but increased the surface roughness. Ultrasound-generated cavitation produced micro-jet, shearing force, and local heating, causing pores, cracks, and depressions on the surface of the banana starch. These findings agree with other investigations of sweet-potato starch and maize starch [53,54]. After complexing with resveratrol, the surface of the starch became rougher and was affected by hydrothermal treatment during the preparation of the starch–resveratrol complexes. Furthermore, the attachment of the white substance indicates that resveratrol was firmly complexed with the starch. We could see partial resveratrol on the surface of the starches (in the black circles).

### 3.7. In Vitro Release Properties

**Figure 8:**
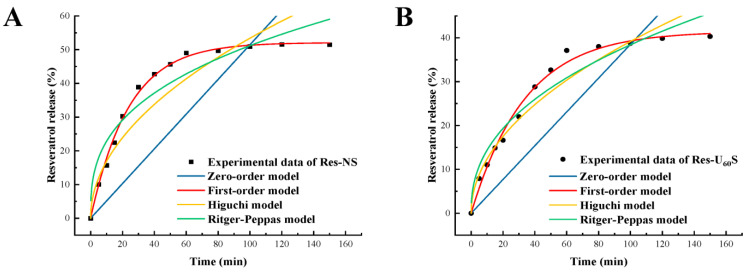
In vitro resveratrol-release profile from Res-NS (**A**) and Res-U_60_S (**B**) during intestinal digestion.

In vitro release behaviors of resveratrol from the starch–resveratrol complexes were evaluated using a dialysis method under conditions that simulated the digestive tract (Figure 8). The fitting results of different kinetic-release models of resveratrol from Res-NS and Res-U_60_S under intestinal conditions are summarized in Table 4. With the prolonging of the digestion time, the resveratrol released from two starch samples increased rapidly at the beginning, then decreased markedly, and reached a plateau state in the end. The cumulative release rates of resveratrol from Res-NS and Res-U_60_S after 150 min in the intestinal fluid were 51.48% and 40.32%, respectively. The lower release rate of resveratrol from Res-U_60_S could be accounted for by the more uniform structure of lowered digestibility. Thus, Res-U_60_S had good retention and sustained release of resveratrol [27,28]. Based on Table 4, compared with the zero-order kinetic equation, Higuchi equation, and Ritger–Peppas equation, the results of Res-NS and Res-U_60_S were found to be more consistent with the first-order model (R^2^ > 0.99). Additionally, the release rate was proportional to the time. This indicates that the diffusion was caused by the relaxation of the starch structure driven by distance [54].

## 4. Conclusions

This work researched the effects of modification by combination of ultrasound and resveratrol treatments on the physicochemical properties and in vitro digestibility of modified banana starch. The ultrasound and resveratrol enhanced the strength of the gel and the viscoelasticity (G′ and G″), respectively. The banana starch was complexed with resveratrol through non-covalent interactions, resulting in the modified starch having a more compact structure and higher thermal stability. Additionally, after the modification, the degree of hydrolysis of the modified banana starch was reduced, whereas the contents of RDS and RS increased as the content of SDS decreased. Therefore, this new modification method could be beneficial for controlling starch structure and digestion behavior. The modified starch could be beneficial to the application of antioxidant phenolic and resistant starch.

## Figures and Tables

**Table 1:** Thermal parameters from DSC thermograms of different samples.

Samples	T_o_ (°C)	T_p_ (°C)	T_c_ (°C)	ΔH (J/g)
NS	64.10 ± 0.26 ^d^	71.14 ± 0.19 ^d^	73.92 ± 0.20 ^d^	1.73 ± 0.14 ^d^
U_60_S	64.82 ± 0.13 ^c^	71.72 ± 0.16 ^c^	74.30 ± 0.22 ^c^	2.36 ± 0.07 ^b^
Res-NS	68.17 ± 0.23 ^b^	74.74 ± 0.13 ^a^	77.17 ± 0.19 ^a^	2.12 ± 0.11 ^c^
Res-U_60_S	70.77 ± 0.16 ^a^	73.99 ± 0.21 ^b^	75.83 ± 0.15 ^b^	2.92 ± 0.10 ^a^

Values are the mean ± standard deviation of three replicates. Means with different letters within each column are significantly different (*p* ≤ 0.05).

**Table 2:** Power-law parameters of starch samples.

Samples	K (Pa·s^n^)	n	R^2^
NS	7.211 ± 0.080 ^c^	0.572 ±0.003 ^a^	0.993
U_60_S	7.582 ± 0.015 ^b^	0.566 ± 0.008 ^a^	0.985
Res-NS	7.545 ± 0.073 ^b^	0.568 ± 0.005 ^a^	0.977
Res-U_60_S	9.446 ± 0.088 ^a^	0.372 ± 0.008 ^b^	0.960

Values are the mean ± standard deviation of three replicates. Means with different letters within each column are significantly different (*p* ≤ 0.05). K is the consistency coefficient (Pa·s^n^), and n is the flow-behavior index.

**Table 3:** Kinetic parameters and starch fraction of simulated GIT digestion of different samples.

Samples	k (×10^−2^/min)	C_∞_ (%)	RDS (%)	SDS (%)	RS (%)
NS	1.57 ± 0.30 ^b^	44.12 ± 0.65 ^a^	32.29 ± 0.29 ^b^	11.40 ± 0.16 ^a^	56.31 ± 0.13 ^c^
U_60_S	1.16 ± 0.19 ^b^	42.82 ± 0.19 ^b^	32.88 ± 0.32 ^ab^	9.93 ± 0.13 ^b^	57.19 ± 0.19 ^b^
Res-NS	1.42 ± 0.24 ^b^	43.51 ± 0.10 ^b^	33.69 ± 0.44 ^a^	9.83 ± 0.52 ^b^	56.48 ± 0.10 ^c^
Res-U_60_S	2.69 ± 0.76 ^a^	40.25 ± 0.16 ^c^	33.74 ± 0.28 ^a^	7.60 ± 0.44 ^c^	58.75 ± 0.16 ^a^

Values are the mean ± standard deviation of three replicates. Means with different letters within each column are significantly different (*p* ≤ 0.05). C_∞_, equilibrium constant; k, kinetic constant; RDS, rapidly digestible starch; SDS, slowly digestible starch; RS, resistant starch.

**Table 4:** Fitting results of the kinetic-release model of resveratrol from Res-NS and Res-U_60_S.

Samples	Models	Fitting Equations	R^2^
Res-NS	Zero-order	Q_t_ = 0.0052 × t	0.1145
	First-order	Ln(1 − Q_t_) = 0.0418 × t − 0.5227	0.9951
	Higuchi	Q_t_ = 0.0536 × t^1/2^	0.8453
	Ritger–Peppas	LnQ_t_ = 9.8287 × Lnt − 0.3517	0.9063
Res-U_60_S	Zero-order	Q_t_ = 0.0039 × t	0.4442
	First-order	Ln(1 − Q_t_) = 0.0293 × t − 0.4179	0.9902
	Higuchi	Q_t_ = 0.0394 × t^1/2^	0.9324
	Ritger–Peppas	LnQ_t_ = 18.5753 × Lnt − 0.4282	0.9402

## Data Availability

Data are contained within the article.
